# Evaluation of Mechanical Properties of 3D-Printed Polymeric Materials for Possible Application in Mouthguards

**DOI:** 10.3390/polym15040898

**Published:** 2023-02-11

**Authors:** Maciej Trzaskowski, Katarzyna Mańka-Malara, Agata Szczesio-Włodarczyk, Jerzy Sokołowski, Jolanta Kostrzewa-Janicka, Elżbieta Mierzwińska-Nastalska

**Affiliations:** 1 Department of Prosthodontics, Medical University of Warsaw, ul. Binieckiego 6, 02-097 Warsaw, Poland; 2 University Laboratory of Materials Research, Medical University of Lodz, Pomorska 251, 92-216 Łódź, Poland

**Keywords:** injury prevention, teeth trauma, martial arts, combat sports, prosthodontics, dental materials, CAD CAM technologies, stereolithography, digital workflow, digital dentistry

## Abstract

Custom mouthguards are used in various sports disciplines as a protection for teeth, temporomandibular joints, and soft tissues of the oral cavity from impact forces. The purpose of this research was to evaluate the mechanical properties of flexible polymeric 3D-printable materials and to select a material with the most favourable physical properties for making intraoral protectors. Four 3D-printable polymeric materials were selected for the evaluation: IMPRIMO LC IBT (Scheu-Dental, Iserlohn, Germany), Keyortho IBT (EnvisionTEC, Gladbeck, Germany), IBT (Formlabs, Somerville, MA, USA), and Ortho IBT (NextDent, Utrecht, Netherlands). A total of 176 samples (44 from each material) was 3D-printed using the stereolitography (SLA) technique. Tensile strength, flexural strength, notch-toughness, Shore hardness, sorption, and solubility tests were conducted. The materials were compared using a series of analyses of variance (one-way ANOVA) with Bonferroni post hoc tests. Statistical analyses were performed with the use of IBM SPSS Statistics 28.0.0 software (IBM, New York, NY, USA). Each material was assigned a score from 1 to 4 depending on the individual test results, and tests were given indexes according to the significance of the parameter in the mouthguard protective function. The number of points obtained by each material in each test was then multiplied by the test index, and the results were tabulated. The material with the highest result among the ones studied—most suitable for the application in mouthguard fabrication—was Keyortho IBT from EnvisionTEC.

## 1. Introduction

Custom mouthguards provide protection for the stomatognathic system from the impact forces, maintaining adequate retention and comfort for its user [1,2,3,4,5,6]. Polymeric materials and polymeric films are, in the last decade, widely used in dentistry and in preventive and restorative therapies due to their mechanical and biological properties [7,8,9]. There are various materials used in mouthguard fabrication: polyvinylacetate-polyethylene or poly(ethylene-co-vinyl acetate) (EVA copolymer), polyvinylchloride, latex rubber, acrylic resin, and polyurethane [10,11]. The main feature of this type of intraoral appliance is to dissipate impact forces and to separate the teeth from soft tissues in case of an injury, thus a material used in their fabrication should have high damping properties, flexibility, low hardness for ease of application, biocompatibility, low fluid sorption, and resistance for regular usage. EVA vacuum-formed custom mouthguards are currently the gold standard in the fabrication of protective splints [12,13,14]. Unfortunately, there are problems with maintaining the correct dimensions of the final splint during thermoforming [15,16,17,18]. Many authors have described methods to prevent the thinning, but the problem remains unsolved [15,16,18]. Currently, mouthguards may also be prepared using thermal injection or flasking using acrylate hybrid composites [3,6,12,19], but the materials and equipment used in such procedures are difficult to procure.

Digital techniques change the possibilities of the stomatognatic system rehabilitation [20,21]. The use of 3D-printing in mouthguard manufacturing could make it possible to design a single protective splint, which can be printed and duplicated while maintaining its dimensions [22]. This is an important feature because intraorally used appliances should be replaced regularly due to hygiene issues [4,23,24,25,26,27]. Additionally, according to Kiatwarawut et al. [28], standards for adequate cleaning of thermoplastic appliances still have to be established. There have been few promising studies published on this subject. Pinho et. al. [29] compared the mechanical behaviour of multi-material samples that were produced using a double-nozzle 3D-printer. The best results were achieved using the ABS-TPU-ABS combination of materials due to the fact that they have the highest resilience value. Saunders et. al. [30] assessed the energy dissipation of 3D-printable material (Arnitel ID 2045 Natural; DSM, Heerlen, The Netherlands), showing that the printed samples dissipated 25% more energy than EVA in both a medium and high strain rate. Liang et. al. [31] proposed a 3D-printing wearable personalised oral delivery device in the form of a mouthguard using fused filament printing (FFF). This method is cheap and fast; however, the final products have a high porosity and an uneven surface [32]. In view of problems with mouthguards’ microbiological contaminations [33,34,35], it should be considered whether this method should be applied given the current level of printing accuracy. Mouthguards were also produced using computer-aided design and manufacturing (CAD/CAM) with polyetheretherketone (PEEK) [36] or 3D-printing using photopolymerizable composite [37].

In dentistry, a common method of 3D-printing is stereolithography (SLA), which gives highly accurate and smooth surfaces [38,39,40]. This technique uses a scanning laser to build parts one layer at a time using light-cured photopolymer resin. The advantage of this method is the possibility to produce high-resolution objects and create complex shapes with undercuts, such as dental devices [41,42]. This technology also does not involve complex material preparation for the printable inks or complex post-treatment of printed appliances. However, the use of liquid resin makes the printing process slower than fused deposed modelling (FDM), in which the liquefied filament is extruded from a nozzle [43]. The purpose of the conducted research was to evaluate the mechanical properties of materials for resin 3D-printing and to select a 3D-printing material with the most favourable physical properties for mouthguard fabrication. The null hypothesis of the analysis was that there are no statistical differences between the compared materials.

## 2. Materials and Methods

### 2.1. Material Selection and Sample Preparation

Based on the inclusion criteria, (elasticity, biocompatibility of resins dedicated for intraoral application), four materials applied in 3D-printing were selected: IMPRIMO LC IBT (Scheu-Dental, Iserlohn, Germany), Keyortho IBT (EnvisionTEC, Germany), IBT (Formlabs, Somerville, MA, USA), and Ortho IBT (NextDent, Utrecht, The Netherlands) (Table 1).

**Figure A1:**
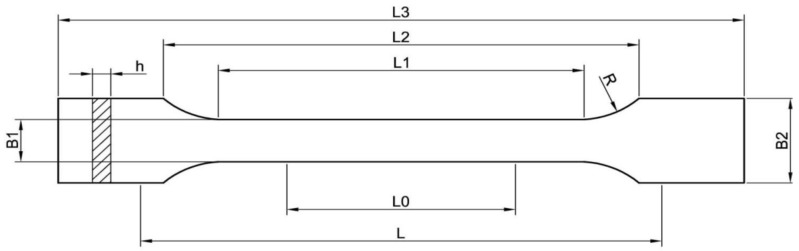
Shape of samples for tensile strength test: L3—total length (150 mm); L2—106 mm; L1—40 mm; R—radius of the curvature (60 mm); B1—width of the shorter part (10 mm), B2—width at ends (20 mm); H—thickness (4 mm); L0—gauge length (50 mm); L—initial distance between holders (115 mm).

Samples for each test were designed in Tinkercad software, which is a free online 3D modeling programme. For notch toughness, the samples were prepared according to the norm: PN-68/C-89028-cuboid in shape with specified dimensions of 15 mm × 10 mm × 3.5 mm. The samples had a notch that was placed on the wall of the impact side. The notch was located at a height of 5.5 mm from the base; it reached up to 1/3 of the thickness of the sample (1.17 mm). It had the shape of an equilateral triangle with a base of 0.8 mm and a height of 1.17 mm with a rounded apex. The samples for flexural strength were prepared according to the ISO 1567:1999 standard, in a cuboid shape with specified dimensions of 64 mm × 10 mm × 3.3 mm. Samples for the tensile strength test were dog-bone shaped (Figure A1). Samples for Shore hardness were prepared according to the norm 638 D2240 and had 10 mm of thickness. Samples for sorption and solubility were prepared according to the ASTM D570 standard, which were cylindrical, with a diameter of 60 mm and a thickness of 3 mm. The number of samples prepared for tests was based on previously published studies [20,44,45,46,47].

**Figure A2:**
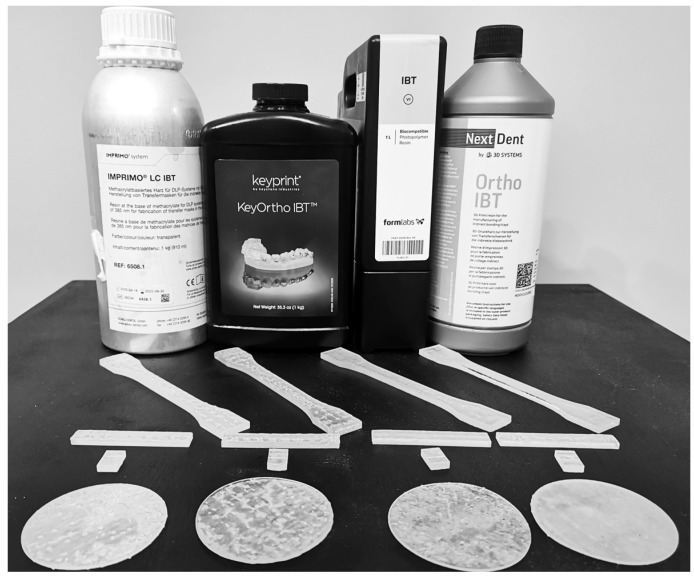
Types of samples from evaluated materials prepared for the research.

Samples were set in the programme at a 45° angle to the base, to enable the free flow of the resin during polymerisation and saved in STL format. The designs were placed in Photon Workshop V2.1.23.RC8 (Anycubic, Shenzen, China) software, where the supports were also designed, and saved in a format supported by the printer. Using a Photon Mono printer (Anycubic, Shenzen, China), samples were printed—the first 10 layers of print (constituting the final bottom layer) had the layer height set at 0.1 mm, exposure time 40 s, retract distance 6 mm, and lifting speed 4 mm/s. The following layer settings were layer height 0.1 mm, exposure time 13.5 s, and lifting speed 9.5 mm/s. After printing, the samples were released and placed in a 10-min bath in 99% isopropyl alcohol using a Wash&Cure 2.0 (Anycubic, Shenzen, China) device, and subjected to a 10-min curing process with a UV light source with a wavelength of 405-nm using the same device. Samples were placed on a 360° rotating platform. After removing the supports, 176 samples were obtained, −44 of each material (Figure A2).

### 2.2. Tensile Strength

**Figure 1:**
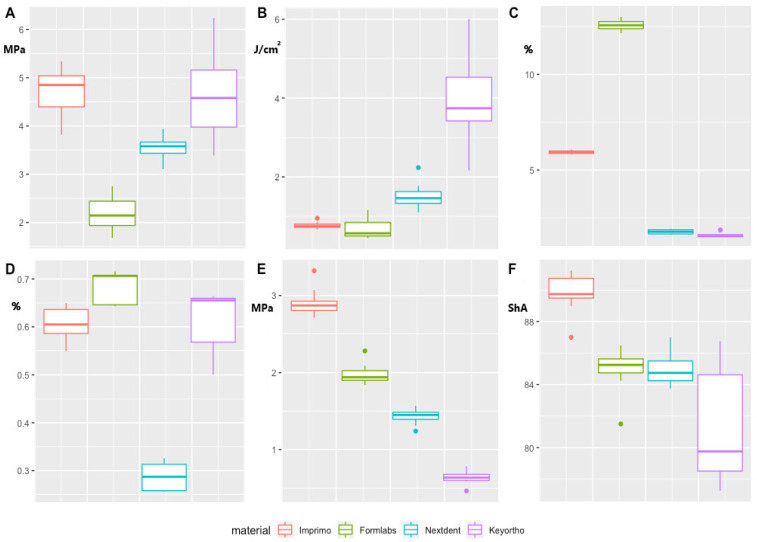
Results of: (**A**)—tensile strength, (**B**)—notch-toughness, (**C**)—sorption, (**D**)—solubility, (**E**) —flexural strength, (**F**)—Shore hardness.

Testing was performed on the basis of the PN-EN ISO 527:1998 standard. A total of 11 measurements was made for each material. Dog-bone samples were used for this study (Figure 1). A ZwickRoell Z020 universal testing machine (ZwickRoell, Ulm, Germany) was used for the tests. The test speed was 5 mm/min.

### 2.3. Notch-Toughness

The test was performed on the basis of the PN-68/C-89028 norm. A total of 11 notched samples was prepared for each study group. The Charpy method was used (HIT5.5P, ZwickRoell, Ulm, Germany). The notch-toughness value was calculated (Equation (1)).

(1) $$\mathrm{NT}\;=\frac{A}{b\times h}$$

where NT—notch-toughness [J/cm^2^], A—absorbed energy [J], b—the width of the specimen at the notch [cm], and h—thickness of the specimen at the notch [cm].

### 2.4. Sorption and Solubility

The test design was based on the ASTM D570 standard. Five cylindrical specimens were prepared for each tested material. The samples were conditioned for 24 h at 50 °C, and after cooling, they were weighed (dry weight—m_d_). Next, samples were put into water at room temperature. The samples were removed from the water after one month; all surface water was wiped off with a dry cloth and weighed (sorbed weight—m_s_). After one month, samples were reconditioned for 24 h at 50 °C, and after cooling, the specimens were weighed (reconditioned weight—m_r_). Sorption (Ab) and solubility (Ds) were calculated (Equations (2) and (3)).

(2) $$\mathrm{WS}\;\left[\%\right]=\frac{m_{s}-m_{d}\;}{m_{d}}$$

(3) $$S\;\left[\%\right]=\frac{m_{d}-m_{r}\;}{m_{d}}$$

### 2.5. Flexural Strength

The test was performed using the three-point bending method on the basis of ISO 1567:1999 standard. A total of 11 measurements using rectangular samples was made for each tested material. The traverse speed was 5 mm/min. Tests were performed using a ZwickRoell Z020 universal testing machine (ZwickRoell, Ulm, Germany). The test was completed after reaching 10% deformation. The strength value was calculated based on maximum force applied during the test (Equation (4)).

(4) $$\mathrm{FS}\;=\frac{3\mathrm{FL}}{2{\mathrm{wd}}^{2}}$$

where FS—flexural strength [MPa], F—maximum force, L—distance between supports, w—the width of the sample, and d—the thickness of the sample.

### 2.6. Shore Hardness

The hardness test was performed using the digital durometer (LX-A, Huatec Group Corporation, Beijing, China), with constant load test stand (TI-D, Sauter -KERN & SOHN GmbH, Ballingen, Germany), using the Shore A scale on the basis of ASTM D2240 standard. A total of 11 samples of each material was printed with a thickness of 10 mm. Measurements were taken at 2 locations on each sample with an LX-A-type hardness tester. The highest measurement was recorded.

### 2.7. Statistical Analysis and Material Comparisons

The four materials were compared with the use of a series of analyses of variance (one-way ANOVA) with Bonferroni post hoc tests. Statistical analyses were performed with the use of IBM Corp. Released 2021. IBM SPSS Statistics for Windows version 28.0.0 software (IBM Corp. Armonk, NY, USA). In the analyses, we considered *p*-values < 0.05 as significant. The number of samples used in each analysis varied between five for sorption and solubility to eleven for flexural strength. For each comparison, descriptive statistics are presented along with the results of the ANOVA. 

## 3. Results

### 3.1. Tensile Strength Results

The scores in the tensile strength test differed significantly (F (3,39) = 48.12, *p* < 0.001) (Table 2; Figure 1). The highest value was achieved by the Imprimo and Keyortho materials, and the difference between them was not statistically significant. The lowest strength was achieved by Formlabs. All post hoc differences were significant at the level *p* < 0.001.

### 3.2. Notch-Toughness Results

During the notch-toughness strength test, not all samples cracked nor did brittle fracturing of samples occur. Eight samples made from the Formlabs and Nextdent material cracked, while from Keyortho and Imprimo material all cracked. The Keyortho material absorbed the energy of a 2 J hammer blow without leading to cracking but only to the appearance of crack propagation. The scores differed significantly (F (3,35) = 23.96, *p* < 0.001). The highest result among the ones studied was achieved by the Keyortho material (*p*-values < 0.001); the results of the other materials were not significantly different from each other (*p*-values higher than 0.060) (Table 3; Figure 1).

### 3.3. Sorption and Solubility Results

The results of sorption of the four materials differed significantly (F (3,16) = 3417.96, *p* < 0.001). In particular, Formlabs scored higher than all other materials (*p*-values < 0.001). Imprimo scored lower than Formlabs and *higher* than Nextdent and Keyorto (*p*-values < 0.001). Nextdent and Keyorto did not differ significantly (*p* = 1.00). (Table 4; Figure 1). For solubility, scores differed significantly (F (3,16) = 67.48, *p* < 0.001) (Table 5; Figure 1). In particular, Nextdent scored lower than all other materials (*p*-values < 0.001). The other materials did not differ from each other (*p*-values higher than 0.117). The lowest value was obtained by the Keyortho material, and the highest sorption result was obtained by the Formlabs material at over 12%.

### 3.4. Flexural Strengh Strength

As a result of the flexural strength test, none of the specimens cracked. The material with the highest strength is Imprimo, which achieved 10% strain at a value of 2.9 MPa (Table 6; Figure 1). Lower values were achieved by Formlabs and Nextdent. The lowest value was achieved by the Keyortho material at 0.64 MPa. All post hoc differences were statistically significant. The results differed significantly (F (3,40) = 645.13, *p* < 0.001). All post hoc differences were significant at the level *p* < 0.001.

### 3.5. Shore Hardness Results

In Shore hardness, test results differed significantly (F (3,40) = 142.84, *p* < 0.001). In particular, the Imprimo material had higher hardness than all other materials (*p*-values < 0.001). The Imprimo material had the highest hardness, Keyortho achieved the lowest value (*p*-values < 0.001), and Nexdent and Formlabs achieved intermediate results that were not significantly different from each other (Table 7; Figure 1).

### 3.6. Materials Comparison

To recommend the best material for 3D-printed mouthguards from those tested, a comparison of achieved results from the perspective of this application was conducted. Each material was given from 1 to 4 points depending on the individual test results. The preferable results for mouthguard materials were high impact strength, bending strength, and tensile strength—the material with highest result among the ones studied in this test was given first place and 4 points, and subsequently lower results were on the following places (1st place—4 points; 2nd—3 points; 3rd—2 points; 4th—1 point). Low material hardness, sorption, and solubility were also preferable properties, and thus, the material with the lowest result was given first place and 4 points. Additionally, the types of tests were assigned indexes according to the significance of the effect of the tested parameter on the properties of the mouthguard. The impact strength score was considered the most important and had an index of 3, bending and tensile strength had index of 2, and the test of hardness, sorption, and solubility had an index of 1. The final score of the material was calculated by multiplying the index of the test and the number of points obtained. The highest score was achieved by Keyortho IBT from EnvisionTEC (Table 8).

## 4. Discussion

The main feature of the mouthguard is its ability to absorb energy and reduce transmitted forces when impacted [44]. Their performance depends on the material used, the thickness, and the inclusion of air cells [7,8,12,13,19,24,47,48]. According to the literature, the optimal thickness at the labial and buccal surface of a mouthguard should be 4 mm. The EVA material having a thickness of 3 mm transmits more than twice the force that passes through the 4 mm material when impacted with the same force [47]. The shape of the product should provide adequate protection without excessive thickness, which may impair the stomatognathic system function [49,50]. The results of the current study show that the polymeric materials have properties that may be favourable in mouthguard fabrication. The high result achieved in the notch-toughness (3.90 ± 1.06 J/cm^2^) and tensile strength test (4.65 ± 0.90 MPa) by the Keyortho IBT material means that it is best suited to absorb the impact energy of the tested samples. It had the lowest result of flexural strength among tested materials. However, none of the samples cracked during the test, and thus, it should not be considered a clinical disadvantage especially because, during usage, mouthguards are not subjected to significant deflections. Applied methodology has not previously been used in the literature for the comparison of mouthguard materials. The dimensions of samples were adjusted to imitate clinically used products—the minimum thickness of EVA providing favourable protective properties while ensuring comfort for a user is 3 mm [51,52,53]. However, there are studies in which a different thickness was used; McNair et al. [54] compared properties of 6 mm samples. ASTM F697-16 guidelines for design and use of mouthguards does not specify their thickness, only the area they should cover.

The physical properties of materials used in mouthguard fabrication have been previously studied by many authors. The shock absorption capacity, which can be defined as the reduction in impact energy of force transmitted through the mouthguard, was compared for different mouthguard materials [7,8,12,13,19,23,24,47,55,56,57,58]. The most common method used to evaluate this parameter was the pendulum or dropped weight, which directly impacted the samples of the material. The range of shock absorption differed depending on the thickness of the sample, the material used, and its chemical composition. In the current study, the applied methodology to calculate the energy absorption was a notch-toughness test, which makes previous results difficult to compare and can be considered a limitation of this research. In this method, the energy absorption is calculated in the presence of the flaw. The ideal surface of the protective splint should be flat and smooth. However, clinically, the occlusal surface of the mouthguard imitates the labial side of the incisors; therefore, it is never an ideal flat surface. Additionally, the elastic polymers used currently in mouthguard fabrication are shown to have cracks, roughness, and increased hardness after usage, so the real conditions in which mouthguards work are closer to the chosen research method [35,59,60]. It would be beneficial to compare the achieved results with other methods used in the literature, such as impact force damping using a Charpy impact hammer. Further studies should also include the comparison of a new material to the current gold standard—poly(ethylene-co-vinyl acetate)—to directly compare its properties.

There is no uniformity in the methodology of compressive and tensile strength of mouthguards as described in the literature. The tear strength of mouthguard materials was determined in a study by Gould et al. [46] based on the standard ASTM D624-0 measuring the force of a complete rupture of a sample. Jagger et al. [61] used samples measuring 50 mm × 10 mm × 2 mm using modification of ASTM D-624 standard. Additionally, McNair et al. [54] used the ASTM D624-00 standard in their research. Paradowska-Stolarz et al. [20] conducted research comparing the compression and tensile modulus of two rigid resins used in 3D-printing using PN-EN ISO 604:2003 norm for axial compression test and PN-EN ISO 527-1-2019 for tensile test. The median tensile strength of the evaluated materials was between 2.17 ± 0.37 MPa for Formabs to 4.72 ± 0.47 MPa for Imprimo. This parameter was previously analyzed for different materials by Going et al. [7]. The tensile strength of 25 mm EVA was between 3 to 20 MPa (mean 11 ± 5). However, they used different parameters and devices, so the achieved results are difficult to compare. The Tinius Olsen Tensile Tester was used according to the method of ASTM D638, samples had dimensions of 3 × 5 × 3/32 inches, and a strain rate was set at 10 inches a minute [7].

Evaluated 3D-printable resins had the median Shore hardness between 81.09 ± 3.56 (Keyortho IBT) and 89.89 ± 1.22 (Imprimo). Similar results were previously published in studies evaluating the properties of mouthguard materials. Going et al. [7] described that 25 mm EVA samples had the median hardness of 83 ± 4. According to Gawlak et al. [12], the median hardness of the single 5 mm plate EVA material (Erkoflex, Erkodent, Pfalzgrafenweiler, Germany) was 74.25 (SD = 1.05), and the most recommended material due to its energy absorption properties, the pressure-injected vinyl polymer (Corflex Orthodontic, Pressing Dental, San Marino, San Marino), had 71.50 (SD = 1.79).

The median water sorption of tested polymers after one month was between 1.61 ± 0.15% for Keyortho IBT to 12.58 ± 0.32% for Formlabs. Previous studies showed that the result of the pressure-injected vinyl polymer was 0.072%, and a 5 mm plate of EVA was 0.134% [9]. Going et al. [7] showed a 0.13%–2.07% (mean 0.48 ± 0.37) of water sorption after 24 h incubation of 25 mm EVA samples. However, there is a significant difference in the applied methodology in those studies because the samples were weighed after 24 h of incubation. In the present study, we decided on one month of exposure to assess total sorption of tested materials. This approach may better characterise the behaviour of materials in long-term use. The 1.61% sorption of the Keyortho IBT samples after this period is clinically acceptable.

Most of the evaluated materials had a high percentage of solubility. The protective splint remains in the oral cavity environment during training and the influence of saliva cannot be omitted as mouthguards are mostly used during sports activities. With the increase in CO_2_ in the blood owing to the increase in physical load, the pH of saliva decreases [62]. Additionally, with the increase in oral cavity temperature, the material comes closer to the glass transition temperature (TG), leading to deformations at lower stress levels [63,64]. In the current study, the lowest median solubility was observed for Nexdent samples—0.29 ± 0.03%, and the highest for Formlabs—0.68 ± 0.04%. Despite the fact that the simplified in vitro test conducted is a limitation of the present study, it should be stated that due to the achieved results the durability and stability of the physical properties of tested materials should be further considered.

The main limitation of this study is that tested materials have not been compared with the most clinically used EVA due to the difficulty to prepare the same dimensions of samples. For the application in mouthguard formation, this material is bought in the form of plates, which are used for thermoforming. To verify whether tested materials have properties enabling their use in such an application, we decided not only to compare the achieved results with those published in the literature but also to compare the resins while paying special attention to the combination of properties that the mouthguard material should have. The introduction of indexes and the ranking of the results from each test was used to choose a material that may be used in further studies. However, subjectively attributed indexes may be considered a limitation of such methodology. During the 3D-printing process a polymer solution may be transformed into the 3D structure via crosslinking, which significantly influences the mechanical and physicochemical characteristics of the material [65]. As the effect of crosslinking reactions is more pronounced on the extrusion-based bioprinting methods [65], crosslinking of PMMA results in increased tensile strength, mechanical stability, elasticity, and solvent resistance [66]. Further research should also consider the relation between the acrylate matrix of the material and overall performance.

## 5. Conclusions

Under the conditions of the current study, it can be stated that the resin polymers for 3D-printing may be considered for application in mouthguard fabrication. The null hypothesis was rejected—there were statistically significant differences between tested materials. The most favorable properties, due to the high notch-toughness and tensile strength as well as low Shore hardness and sorption, were found in the Keyortho IBT (EnvisionTEC, Gladbeck, Germany) material. Further studies determining the clinical usage of this material in mouthguards fabrication as well as comparison with the currently used materials should be conducted to determine whether it can be used in this application.

## Figures and Tables

**Table 1:** Composition of materials used in the research.

Material	Manufacturer	Composition
IMPRIMO LC IBT	Scheu-Dental, Germany	Bisphenol A Ethoxylate Dimethacrylate, Diurethane dimethacrylate, Isobornyl methacrylate, Tetraethylene glycol dimethacrylate, Phenylbis(2,4,6-trimethylbenzoyl)phosphine oxide
Keyortho IBT	EnvisionTEC, Germany	Methacrylate Monomers, Acrylate Monomer
IBT	Formlabs, USA	Dimethacrylate Oligomer, Urethane Dimethacrylate
Ortho IBT	NextDent, Netherlands	Aliphatic Difunctional Urethane Acrylate, Bisphenol A ethoxylate dimethacrylate, Hexyl Methacrylate, Phosphine oxide

**Table 2:** Statistical analysis of tensile strength results.

Material	N	M	SD	Min	Max	M ± SD
Imprimo	11	4.72	0.47	3.82	5.34	4.72 ± 0.47
Formlabs	10	2.17	0.37	1.68	2.75	2.17 ± 0.37
Nextdent	11	3.54	0.23	3.11	3.94	3.54 ± 0.23
Keyortho	11	4.65	0.90	3.39	6.24	4.65 ± 0.90

Formlabs < Nextdent < Keyorto = Imprimo.

**Table 3:** Statistical analysis of notch-toughness results.

Material	N	M	SD	Min	Max	M ± SD
Imprimo	12	0.77	0.08	0.67	0.95	0.77 ± 0.08
Formlabs	8	0.70	0.27	0.45	1.16	0.70 ± 0.27
Nextdent	8	1.52	0.36	1.10	2.24	1.52 ± 0.36
Keyortho	11	3.90	1.06	2.16	6.01	3.90 ± 1.06

Keyorto > Nextdent = Formlabs = Imprimo.

**Table 4:** Statistical analysis of sorption results (n = 5).

Material	M	SD	Min	Max	M ± SD
Imprimo	5.94	0.10	5.83	6.07	5.94 ± 0.10
Formlabs	12.58	0.32	12.17	13.01	12.58 ± 0.32
Nextdent	1.77	0.13	1.62	1.93	1.77 ± 0.13
Keyortho	1.61	0.15	1.51	1.87	1.61 ± 0.15

**Table 5:** Statistical analysis of solubility results (n = 5).

Material	M	SD	Min	Max	M ± SD
Imprimo	0.61	0.04	0.55	0.65	0.61 ± 0.04
Formlabs	0.68	0.04	0.64	0.72	0.68 ± 0.04
Nextdent	0.29	0.03	0.26	0.33	0.29 ± 0.03
Keyortho	0.61	0.07	0.50	0.66	0.61 ± 0.07

**Table 6:** Statistical analysis of flexural strength results (n = 11).

Material	M	SD	Min	Max	M ± SD
Imprimo	2.90	0.17	2.71	3.32	2.90 ± 0.17
Formlabs	1.98	0.13	1.84	2.28	1.98 ± 0.13
Nextdent	1.43	0.10	1.24	1.57	1.43 ± 0.10
Keyortho	0.64	0.09	0.46	0.79	0.64 ± 0.09

**Table 7:** Statistical analysis of hardness results (n = 11).

Material	M	SD	Min	Max	M ± SD
Imprimo	89.89	1.22	87.00	91.25	89.89 ± 1.22
Formlabs	85.00	1.34	81.50	86.50	85.00 ± 1.34
Nextdent	84.93	0.95	83.75	87.00	84.93 ± 0.95
Keyortho	81.09	3.56	77.25	86.75	81.09 ± 3.56

**Table 8:** Comparison of evaluated 3D-printable materials depending on the test result and coefficient calculations.

		Imprimo			Formlabs			Nextdent			Keyortho		
	Test Index	Place	Points	Result	Place	Points	Result	Place	Points	Result	Place	Points	Result
Notch-toughness	3	3	2	6	4	1	3	2	3	9	1	4	12
Flexural strength	2	1	4	8	2	3	6	3	2	4	4	1	2
Tensile strength	2	1	4	8	4	1	2	3	2	4	2	3	6
Shore hardness	1	4	1	1	3	2	2	2	3	3	1	4	4
Sorption	1	3	2	2	4	1	1	2	3	3	1	4	4
Solubility	1	2	2	2	3	1	1	1	3	3	2	2	2
	Score			27			15			26			30

## Data Availability

The data presented in the study are available on reasonable request from authors of this article.
